# Exploring the challenges and features of implementing performance-based payment plan in hospitals: a protocol for a systematic review

**DOI:** 10.1186/s13643-021-01657-x

**Published:** 2021-04-17

**Authors:** Asieh Mousaloo, Mehrdad Amir-Behghadami, Ali Janati, Masoumeh Gholizadeh

**Affiliations:** 1 Student Research Committee (SRC), Tabriz University of Medical Sciences, Tabriz, Iran; 2 Iranian Center of Excellence in Health Management (IceHM), Department of Health Service Management, School of Management and Medical Informatics, Tabriz University of Medical Sciences, University Rd, Golbad, EAZN, Tabriz, 5165665811 Iran; 3 Tabriz Health Services Management Research Center, Health Management and Safety Promotion Research Institute, Tabriz University of Medical Sciences, Tabriz, Iran

**Keywords:** Implementation, Performance-based payment, Challenges, Features, Systematic review, Protocol

## Abstract

**Background:**

Implementing performance-based payment (PBP) plan has led to developing a number of significant potentialities such as performance improvement and effectiveness, quality improvement of provided services, and decline in health system expenditure in hospitals. Despite the fact that PBP plan has a variety of potential advantages, its implementation still may face some challenges. Hence, it seems crucial to identify these barriers and challenges in order to devise some strategies and interventions to pave the way for better implementation of PBP in hospitals. The aim of this proposed protocol is to identify, summarize, and synthesize the existing evidence by undertaking a systematic review to explore the challenges, barriers, and features of implementing PBP in hospitals.

**Methods and analysis:**

An inclusive search of the literature will be conducted in seven international and national databases including PubMed/MEDLINE, Scopus, Cochrane Library and Web of Science, Magiran, Scientific Information Database (SID), and Barakat knowledge network system (BKNS). The search will be limited to the studies published in English or Persian language. Database search will be supplemented by hand-search of citation, reference lists, and grey literature sources. Based on the pre-established criteria in all steps of the review, two researchers will independently screen all of the retrieved studies. Any discrepancies will be resolved through a discussion between two researchers. In cases where consensus is not reached, it will be referred to a third researcher. The methodological quality of all the included studies will be appraised using the Mixed Methods Appraisal Tool (MMAT). The data will be extracted by means of using a data extraction form, which will be developed and piloted by the research team. The findings will be synthesized through directed content analysis method.

**Discussion:**

With the growth and development of payment systems all over the world, it is expected that recognizing the challenges of implementing a PBP plan in hospitals will be useful in developing and designing strategies to better implement this plan.

**Systematic review registration:**

PROSPERO registration number CRD42020152569

**Supplementary Information:**

The online version contains supplementary material available at 10.1186/s13643-021-01657-x.

## Background

Payment system is one of the factors affecting service-providers’ behavior and hence service quality in health system [1]. Accordingly, performance-based payment (PBP) plan was devised so as to enhance quality and efficiency and at the same time reduce extra expenses. It also provides the payers and service-providers with the chance of establishing a relationship between their economic motivations and quality of their performance [1]. Due to its potential advantages, PBP is extensively used in health system of a big number of countries. However, strong evidence supporting whether it has succeeded or failed to enhance quality and effectiveness is still inconclusive and at times extremely disparate. It is claimed that much difference and dispersion between designing, performing, and implementing PBP in different systems is one of the main factors of this failure [2].

Considering the fact that, like other organizations, in hospitals also human plays a pivotal role and staff work in accordance with the state regulations and rules, dissatisfaction with salary or payment or unfair and inefficient payment system leads to a number of issues like: staff dissatisfaction, absence, quitting service, complaint, and other similar organizational problems [3, 4]. Therefore, the aim organization managers seek is retention and empowerment of the qualified human resources, which according to the issues related to fair payment system is one of the factors contributing to accomplishment of such an aim [5].

Primary origin of PBP in health care dates back to the late 1990s in the USA private sector. In 2002, a number of PBP plans were active in the private sector in the USA; however, such innovations were mainly miniscule and experimental. The first large-scale PBP plan in the private sector was introduced by the Comprehensive Health Care Association in California in 2003 and still is active. Since then, PBP plans have been employed by strategic health purchasers in most of the countries in order to strengthen and reform traditional payment systems [6]. Nowadays, most organizations in charge of protecting health and Medicaid program resort to PBP plan. The first one was developed by Medicare and Medicaid centers in 2003. After its implementation, a number of positive accomplishments in terms of improved healthcare services were reported. Successful implementation of the plan made policy-makers advocate PBP in 2010 as it followed Patient Protection and Affordable Care Act (PPACA) [7].

Over recent years, PBP plan has been applied as a quality improvement strategy and cost-effective health care in a number of Organizations for Economic Co-operation and Development (OECD) and American countries [8]. Indeed, PBP is a motivation plan, which is devised to orient incentive pays to organizational objectives. To put it in a simple way, in a PBP plan, financial incentives are paid for performance improvement in accordance with certain criteria including quality, efficiency, effectiveness, and/or other measures like justice [9]. Considering the fact that more than a decade has passed since the implementation of PBP as an intervention developed to increase the quantity and quality of health care, it seems that based on this theory, providing financial incentives to health care workers to achieve output goals will motivate them to produce more or better outcomes and thus improve their performance [10].

It is undeniable that payment has a significant effect on motivation of staff and their job performance in any organization. In this vein, psychologists hold the idea that quite a few of any individual’s needs are directly or indirectly met by money [11]. Hence, there is no doubt that money is of the most important incentives. As financial incentives are of the most important factors affecting personal and organizational behavior in health system and also are effective in health system planning and quantity and quality of services [12], it is crucial that when devising a decent payment system the tremendous effects of incentives on customer or seller behavior be taken into account [13].

In PBP plan, health care service providers receive financial incentives based on their score, which may include clinical quality, used resources, and reporting outcomes. Therefore, so as to establish an efficient bond between payment and performance, performance should be appraised through an accurate and valid approach. It is because, if we fail to bind payments with outcomes, there will be a decline in incentive and whereby performance of staff. It is potential that daily payment, lack of clarification, not determining standard obligatory work, extra work, and lack of competency appraisal criteria will lead to staff dissatisfaction and as a result to dissipation of finance and human resources of the organization [10].

Along with spread and development of PBP plan, the number of studies concerning effects of PBP plan has had a rapid increasing trend over the last 15 years [7]. Despite its potential advantages, PBP plan has been usually reported as a problematic one. Therefore, barriers and challenges of implementing PBP plan in hospital setting may be attributed to payment and financing, organizing, rules and regulations, and behavior of policy-makers, senior managers, and providers. A number of systematic reviews, having focused on PBP, have synthesized the available evidence; however, they have reported different results. These reviews included experimental studies, which focused on preventive services and accentuated incentive performance.

PBP plans are complex, and their effects may vary based on design, context, and other implementation processes. Kondo et al. conducted a systematic review and key informant (KIs) interviews to better understand the implementation factors that modify PBP effectiveness [14]. Their findings suggest that PBP plan should be evaluated regularly and poor performance areas should be targeted. In addition, actions and incentives must align with organizational priorities, and programs must be changed over time in response to data and provider input. In a systematic review, Van Herck et al. investigated the effects, design choices, and context of PBP in health care The results showed that the potential effects for specific performance goals ranged from absent or insignificant to strongly beneficial [15].

Baxter et al. conducted a systematic review to better understand the experiences of health care leaders implementing hospital funding reforms in the OECD [16]. They concluded that, regardless of what type of hospital funding reform was implemented, the following should be considered: adequate infrastructure; organizational commitment; human, financial, and information technology resources; change champions; and a personal commitment to quality care. In summary, the literature clearly presents significant challenges, complexities, and ambiguity regarding the implementation of PBP plan. This information is essential for informing the processes and decisions related to implementation of PBP.

Identifying challenges and barriers as well as implications of PBP implementation may eliminate the individual and organizational factors associated with the successful adoption of this funding model and whereby enhance the expected consequences. The evidence from this review will be helpful to leaders, managers, and policy-makers who are responsible for the implementation and maintenance of funding models. Hence, it can be claimed that in terms of acknowledging the evidence on success and failure of the factors effective in implementing performance-based payment program in hospitals [12], identifying and acknowledging barriers and challenges is crucial. It is because it can be useful and functional at the time of designing strategies and interventions to improve efficient application of PBP plan. The aim of the proposed protocol is to perform a systematic review in order to identify, summarize, and synthesize the published literature on challenges, barriers, and features of implementing PBP in hospitals.

## Methods

### Review method

This systematic review protocol will be conducted and reported in accordance with the Preferred Reporting Items for Systematic Review and Meta-analysis Protocols (PRISMA-P) [17] (see Additional file 1) and the results will be reported following the PRISMA Statement guidelines [18].

The summary of this protocol has already been registered in the International Prospective Register of Systematic Review PROSPERO (registration number, CRD42020152569; available at https://www.crd.york.ac.uk/prospero/display_record.php?ID=CRD42020152569).

### Search strategy and information sources

Electronic search will be conducted in seven international and national databases including PubMed/MEDLINE, Scopus, Cochrane Library and Web of Science, Magiran, SID, and BKNS. This process will be performed by a researcher familiar with the search of databases. All of the search will be done with no time limit until the end of August 2020. Thereafter, the snowballing search strategy will be employed to ensure that other eligible studies are identified according to the references lists and citation of the included studies. Also, grey literature sources will be considered, that is, OpenGrey, National Health Service (NHS) evidence, Grey Literature Exploitation (EAGLE), the Healthcare Management Information Consortium (HMIC), ProQuest Dissertations, and IranDoc databases. Relevant keywords identified from the preliminary search will be tested and approved by research team. Based on the key components of the aim of this review and in accordance with the inclusion criteria, the agreed upon keywords will be used to devise the comprehensive search strategy, which consists of a set of search terms including MeSH term and free words. English key words and their Persian equivalents will be used. A list of the search strategy for PubMed has been formulated and will be replicated for the other electronic databases (see Additional file 2)**.**

### Eligibility criteria

This systematic review will screen the published studies that focus on challenges and features of implementing performance-based payment in hospitals.

The eligibility criteria for the study inclusion have been defined using the framework SPIDER (sample, phenomenon of interest, design, evaluation, and research type), which indicates the key components of research questions. It has been developed as a substitute for population, intervention, comparison, outcome, and study type (PICOS) [19] in order to optimize identification of qualitative review question. Each element of it will be included in the search strategy. Similarly, potential alternative search terms will be included to maximize the chances of retrieving relevant studies.

The criteria outlined below will be applied to include and exclude studies:

### Inclusion criteria

Studies that meet all of the following criteria will be included:

- S–sample: all types of hospital (e.g., general and specialized) and care (e.g., emergency, inpatient and outpatient care) in any country
- PI–phenomenon of interest: the studies that focus on challenges and features of PBP implementation in hospitals
- D & R–design and research type:
  - Quantitative studies (e.g., randomized controlled trials, non-randomized controlled trials, cross-sectional study, case-control study or controlled before-after study and survey),
  - Qualitative studies (e.g., phenomenology, narrative research, case study and qualitative description),
  - Mixed method studies (e.g., convergent design, sequential explanatory design and sequential exploratory design)

### Exclusion criteria

Studies that meet any of the following criteria will be excluded:

- The studies that are not part of primary research (such as articles reported to conferences, Commentaries, letter to editor, editorials, opinion, discussion, case reports, reviews, meta-analyses, and other secondary studies.
- The published studies in a language other than English and/or Persian.
- Full-text not available.

### Study selection

The EndNote software package (VX6) will be employed for data management during the review. All the databases will be searched once and then the search results will be exported to a single EndNote software library in order to select and remove duplicate references. Titles and abstracts of all the retrieved studies will be independently double-screened by two researchers. After the first round of screening, the full text of potentially eligible studies will be independently read and assessed based on the predetermined inclusion and exclusion criteria. In cases where the information about the eligibility of a study is incomplete or limited (e.g., when only an abstract is available), we will contact the authors to request the full text or further details. Any potential discrepancies will be resolved through a discussion between the two researchers. In cases where consensus is not reached, it will be reconsidered by a third researcher.

### Quality appraisal

Analyzing and interpreting preliminary studies in a systematic review requires qualitative appraisal and appraisal of susceptibility in biases [20]. The latest version of the Mixed Method Appraisal Tool (MMAT version 2018) will be used to evaluate the quality of all three types of studies including quantitative (quantitative randomized controlled trials, quantitative non-randomized, quantitative descriptive), qualitative, and mixed-method studies (see Additional file 3) [21]. Validity and reliability of the tool have been tested and piloted for all methodologies [22, 23]. The tool comprises two screening questions, five criteria for each type of study that is scored on a categorical scale as either ‘yes,’ ‘no,’ or ‘cannot tell.’ All the included studies will be appraised using the initial two screening questions, which would indicate whether further methodological quality appraisal is feasible or appropriate. If responses to both questions are either ‘no’ or ‘cannot tell’, they will be excluded from further evaluation. The total percentage quality score for each study will be calculated based on the MMAT scoring guide. Only the number of items scored ‘yes’ is summed for an overall score [24]. For the purposes of this review, scores of ≤ 50% will be regarded as ‘low quality,’ while a score in the range of 51–75% as is regarded as ‘average quality.’ A score in the range of 76–100%will be considered as ‘high quality.’ Critical appraisal requires judgment; hence, quality appraisal of the included studies will be independently considered by the two researchers. Potential disagreements will be resolved through reaching a consensus and if needed through consulting a third researcher.

### Data extraction

Data from each of the included studies will be extracted and abstracted based on the characteristics of each publication including: authors, publication year of the study, objective(s) of the study, country of the study, type of study/study design, setting or types of hospital (e.g., general and specialized), subsets of hospitals (e.g., ambulatory vs inpatient or diagnostic vs interventional), language of the study, and MMAT score. The data extraction process will be checked by another researcher who will critically examine the accuracy of the extraction done by the lead researcher. Any disagreements will be resolved through discussion and consultation with a third researcher if required.

### Data synthesis

In terms of the content of publications, especially findings and discussions section, data on the barriers and challenges of implementing PBP will be considered for synthesis. The data will be synthesized through directed content analysis method [25]. This process will be systematically compressed into fewer categories in accordance with explicit coding rules by means of using inductive reasoning. In other words, coding of the identified items relates to the challenges grouped into sub-categories and main categories. Accordingly, the categories will be established following preliminary data reviews.

In this study, directed content analysis is guided by a structured approach, which uses existing theory (or literature/research) to identify the key concepts as initial coding categories. Sometimes, there is existing theory or previous research on a phenomenon that is incomplete or can be further described. We appreciate that existing theory or research can help focus on the research question. It can provide predictions about variables of interest or relationships between variables, whereby it helps to determine the initial coding schedule or relationships between codes [25, 26]. In this systematic review, we will not limit the search to low-income countries or high-income countries. Depending on the number of studies related to the challenges of implementing a PBP, it can be synthesized separately for low-income and high-income countries. It will also lead to comparing the challenges of identified studies based on low-income and high-income countries. The primary researcher will conduct the qualitative data collection and analysis process, and intermittently consult with the secondary researcher on the coding structure and emergent categories. Any disagreements will be resolved through discussion with the third researcher, if required.

### Patient and public involvement

Patients and the public were not involved with the development of this protocol.

## Discussion

While proponents of PBP make great claims about their achievements and potentiality, a review of literature shows that there is little evidence to support the claims [12]. This is mainly due to the fact that PBP evaluation is very difficult. A systematic review done by Eijkenaar et al. indicates that PBP effect in hospitals is still limited and most of the studies done on hospital PBP plans have mainly noticed design qualities like size of incentives or their structure or criteria selection [7]. Two other systematic reviews on PBP effectiveness have been done in two low-income countries. Their results indicate that there is a low chance to establish stable changes in providing health services through such plans. Indeed, there is a dearth of evidence to support performing this plan [8, 27]. Therefore, according to the findings of these studies, it can be concluded that PBP faces a number of challenges. Therefore, it is crucial that some systematic reviews and qualitative studies be performed in order to identify its challenges.

As a comprehensive intervention in a specific and complex system, PBP seeks to improve health sector by changing and re-engineering the organizational structure of health system according to its financing mechanisms, information systems, planning, monitoring, and evaluation. Therefore, in the face of such methodological barriers and resistance to change, initial assessments should be made based on the context (economic, social, political) as well as the content and process of implementation. To the best of our knowledge, identifying the challenges inherent in implementing PBP in these assessments can be useful and effective.

Therefore, the proposed systematic review will explore challenges, barriers, and features that influence the implementation of PBP in hospitals by reviewing, summarizing, and synthesizing the existing literature. Findings of the present study will provide a ground to understand the point whether barriers and challenges of implementing PBP plan in hospitals are changing or they change as time passes. It seems that due to the growth and development of payment systems in time transition, it is likely that other new and different factors will be identified that hinder their implementation. Thus, recognition of this issue will be helpful when developing and designing strategies to implement the best possible PBP plan.

Nevertheless, it is likely that quite a few of the barriers and challenges, which have been identified in the literature, remain unchanged. It is the fact that may reflect the factors that are inherent in implementing PBP plan in hospitals. As this review focuses on barriers, challenges, and features of implementing PBP plan, we are of the opinion that its findings and recommendations will draw the attention of policy-makers, planners, hospital managers, health service providers, and academicians. We will publish findings of the study through dissemination in an international peer-reviewed journal. Also, we will present it at national conferences.

### Strengths and limitations of this study

All steps of the review (screening titles, abstracts and full texts of all of the retrieved studies, assessing the quality of full texts, and extracting data) will be independently done by two researchers. Since the search strategy in bibliographic databases is likely to miss relevant studies, additional studies will be searched in other sources of information for such citation follow-up and reference lists of all the included studies. The points that will restrict the comprehensiveness of this proposed systematic review include excluding the articles written in any language other than English and/or Persian and hence omitting the non-English and/or non-Persian evidence related to the barriers, and challenges inherent in implementing PBP in hospitals.

## Supplementary Information

**Additional file 1.** PRISMA-P 2015 Checklist.

**Additional file 2 **Proposed search strategy (PubMed).

**Additional file 3 **Mixed Methods Appraisal Tool (MMAT) Version 2018.

## Data Availability

Not applicable.

## Abbreviations

PBP: Performance-based payment
SID: Scientific Information Database
BKNS: Barakat knowledge network system
MMAT: Mixed Methods Appraisal Tool
PPACA: Patient Protection and Affordable Care Act
OECD: Organization for Economic Co-operation and Development
PRISMA-P: Preferred Reporting Items for Systematic Review and Meta-analysis Protocols
NHS: National Health Service
HMIC: Healthcare Management Information Consortium
PICOS: Population, Intervention, Comparison, Outcomes, Study design
