# Supplementary material for: Exploring the challenges and features of implementing performance-based payment plan in hospitals: a protocol for a systematic review
Source: Syst Rev. 2021 Apr 17;10:114. doi: 10.1186/s13643-021-01657-x (PMC8052724; doi:10.1186/s13643-021-01657-x)
Supplement: Supplementary file 2 — Additional file 2 Proposed search strategy (PubMed). [file 13643_2021_1657_MOESM2_ESM.docx]

Proposed search strategy (PubMed);

1. “Performance-based pay*” ti, ab.
2. "Performance-based contracting" ti, ab.
3. "Performance-based reimbursement" ti, ab.
4. "Performance-based financing" ti, ab.
5. "Results-based financing" ti, ab.
6. “Result-based payment” ti, ab.
7. "Output-based payment" ti, ab.
8. “Performance-related payment” ti,ab.
9. "Pay for performance" ti, ab.
10. "P4P" ti, ab.
11. "PFP" ti, ab.
12. "PBP" ti, ab.
13. "Pay for value" ti, ab.
14. "Pay for quality" ti, ab.
15. "Payment for quality" ti, ab.
16. "Value-based purchasing" ti, ab.
17. "Incentive reimbursement" ti, ab.
18. "Incentive program" ti, ab.
19. "Quality-based purchasing" ti, ab.
20. "Quality incentive" ti, ab.
21. "Quality Improvement/economics"[Mesh]
22. "Reimbursement, Incentive/economics"[Mesh]
23. "Financing, Government/methods"[Mesh]
24. "Quality Assurance, Health Care/economics"[Mesh]
25. "Non?payment"
26. Compensation ti,ab.
27. Redress ti,ab.
28. Salary ti,ab.
29. “Fringe benefit” ti,ab.
30. Pay* ti,ab.
31. Performance ti,ab.
32. "Financial incentive*" ti, ab.
33. Quality ti, ab.
34. "Monetary incentive*" ti, ab.
35. Bonus ti, ab.
36. reward* ti, ab.
37. Hospital* ti,ab.
38. 26 AND 27
39. 28 AND 29
40. 30 AND 31
41. 32 AND 33
42. 34 AND 33
43. 35 AND 33
44. 36 AND 33
45. 1 OR 2 OR 3 OR 4 OR 5 OR 6 OR 7 OR 8 OR 9 OR 10 OR 11 OR 12 OR 13 OR 14 OR 15 OR 16 OR 17 OR 18 OR 19 OR 20 OR 21 OR 22 OR 23 OR 24 OR 25 OR 38 OR 39 OR 40 OR 41 OR 42 OR 43 OR 44
46. 37 AND 45
